# Novel 3D Mature Osteocyte Perfusion Platform Reveals Subtype-Dependent and Radiation-Sensitive Disruption of Mechanotransduction by Breast Cancer Secretomes

**DOI:** 10.64898/2026.10.22.760275

**Published:** 2026-10-23

**Authors:** Antonio Salcido-Alcántar, Corrin Bowman, Ellie Goldman, Emma N Briggs, Anthony M Bierschenk, Tanvi K Rami, William R Thompson, Maureen E Lynch

## Abstract

Osteocytes (OCys) coordinate bone remodeling by transducing mechanical signals into anabolic cues, primarily through suppressing sclerostin and RANKL. In breast cancer (BCa) bone metastasis, mechanical loading fails to restore lost bone, pointing to tumor-induced OCy mechanosensory dysfunction. Here, we investigate how BCa molecular subtype heterogeneity and frontline therapies like radiation influence OCy mechanobiology. We leveraged a 3D mineralized scaffold paired with a forced perfusion bioreactor to establish a physiologically relevant model of mature, dendrite-bearing OCys. After optimizing perfusion parameters, 3D mature OCys were exposed to conditioned media (CM) from estrogen receptor-positive (ER+; ZR-75-1) or triple-negative (TN; MDA-MB-231) BCa cells generated 24 h post-irradiation (0 vs. 2 Gy). Downstream OCy gene expression ( *Sost, Tnfsf11, Tnfrsf11b* ) and tumor CM proteomic profiles (84 oncology targets) were evaluated. Differentiated 3D MPC-2s exhibited interconnected dendritic networks, classical responsiveness to parathyroid hormone, and robust flow-induced *Sost* suppression. Non-irradiated ER+ BCa CM preserved canonical OCy mechanoresponsiveness ( *Sost* downregulation post-flow), but this response was eliminated when ER+ cells received 2 Gy radiation. Conversely, TN CM blunted flow-induced *Sost* suppression regardless of irradiation status. Proteomic profiling revealed that BCa subtype was the primary driver of secretome composition, whereas 2 Gy radiation induced distinct directional secretome shifts between subtypes. These findings demonstrate that BCa-induced impairment of OCy mechanosensing is subtype-dependent and that low-dose radiation further exacerbates secretome-mediated mechanosensory loss in ER+ disease, highlighting key tumor-derived targets for restoring skeletal health.

